# Clinical utility of the left atrial strain analysis

**DOI:** 10.1007/s12574-025-00695-x

**Published:** 2025-07-03

**Authors:** Katsuji Inoue, Masaru Obokata

**Affiliations:** 1 Department of Community Emergency Medicine, Ehime University Graduate School of Medicine, Ohhira 1-638, Yawatahama, Ehime 796-8502 Japan; 2 Department of Cardiology, Pulmonology, Hypertension and Nephrology, Ehime University Graduate School of Medicine, Toon, Japan; 3 Department of Cardiovascular Medicine, Gunma University Graduate School of Medicine, Maebashi, Japan

**Keywords:** Left atrial strain, Left atrial function, Left ventricular diastolic function, Left ventricular filling pressure, Heart failure with preserved ejection fraction, Exercise stress echocardiography

## Abstract

The importance of left atrial (LA) function is recognized, especially when left ventricular function fails or atrial fibrillation occurs and heart failure (HF) develops. A compensatory mechanism of LA function unremittingly operates to maintain cardiac performance and prevent the occurrence of HF. LA function consists of three components: booster pump, reservoir, and conduit function. Echocardiography enables assessment of these components of LA function by LA volumetric changes in one cardiac cycle. Currently, LA strain has emerged as a valuable diagnostic and prognostic tool in patients with various cardiovascular diseases. An aging society is confronted with an issue of public health due to an increased number of patients with heart failure with preserved ejection fraction (HFpEF). Because effective medical and interventional approaches for patients with HFpEF are limited, early diagnosis and prevention are important to avoid a transition from preclinical HF stage to clinically overt HFpEF. Echocardiography with LA strain analysis and exercise stress echocardiography have the potential to identify patients with undiagnosed HFpEF, thus facilitating several preventive and therapeutic interventions. In this review, the development, measurement, and clinical application of LA strain are summarized and future perspectives in preventive medicine, accurate diagnosis, and therapeutic guidance for patients with HF are discussed.

**Graphical abstract:**

Clinical application of LA strain. A. Mitral inflow patterns and LA strain curves in LVDD. A cutoff value of LA reservoir strain of 35% can differentiate patients with LVDD grade 0 from those with LVDD grades 1–3. Similarly, an LA strain of 24% distinguishes patients with LVDD grades 0–1 from those with LVDD grades 2–3, while an LA strain of 19% serves as a threshold to differentiate LVDD grades 0–2 from grade 3. The percentage values on the LA strain curves represent the LA reservoir strain values corresponding to the white-dotted line. B. LA reservoir strain can be used to estimate LV filling pressure. A cutoff value < 18% for reservoir strain can be used to estimate an elevated LV filling pressure > 12 mmHg. C. LA reservoir strain at rest can be used to diagnose HFpEF. This case was a 75-year-old woman with a history of repeated catheter ablation for AF evaluated for progressive symptoms of exertional dyspnea despite maintaining a sinus rhythm. She had a normal LV ejection fraction of 65% with an enlarged left atrium (LA volume index 57 mL/m^2^) and reduced LA reservoir strain of 13% (left panel). Right heart catheterization showed mildly elevated pulmonary capillary wedge pressure at rest (a-wave, 18 mmHg; v-wave, 24 mm Hg). However, the pulmonary capillary wedge pressure increased during exercise (a-wave, 34 mmHg) with large v-waves (47 mmHg), suggesting severely reduced LA compliance (right panel). D. LA reservoir strain at rest can be used to predict future HF events in patients with preserved LV ejection fraction. Patients with a low LA reservoir strain value ≤ 21% experienced more HF events than those with a high LA strain value > 21%. LA, left atrial; HF, heart failure; LV, left ventricular; DD, diastolic dysfunction; HFpEF, heart failure with preserved ejection fraction.

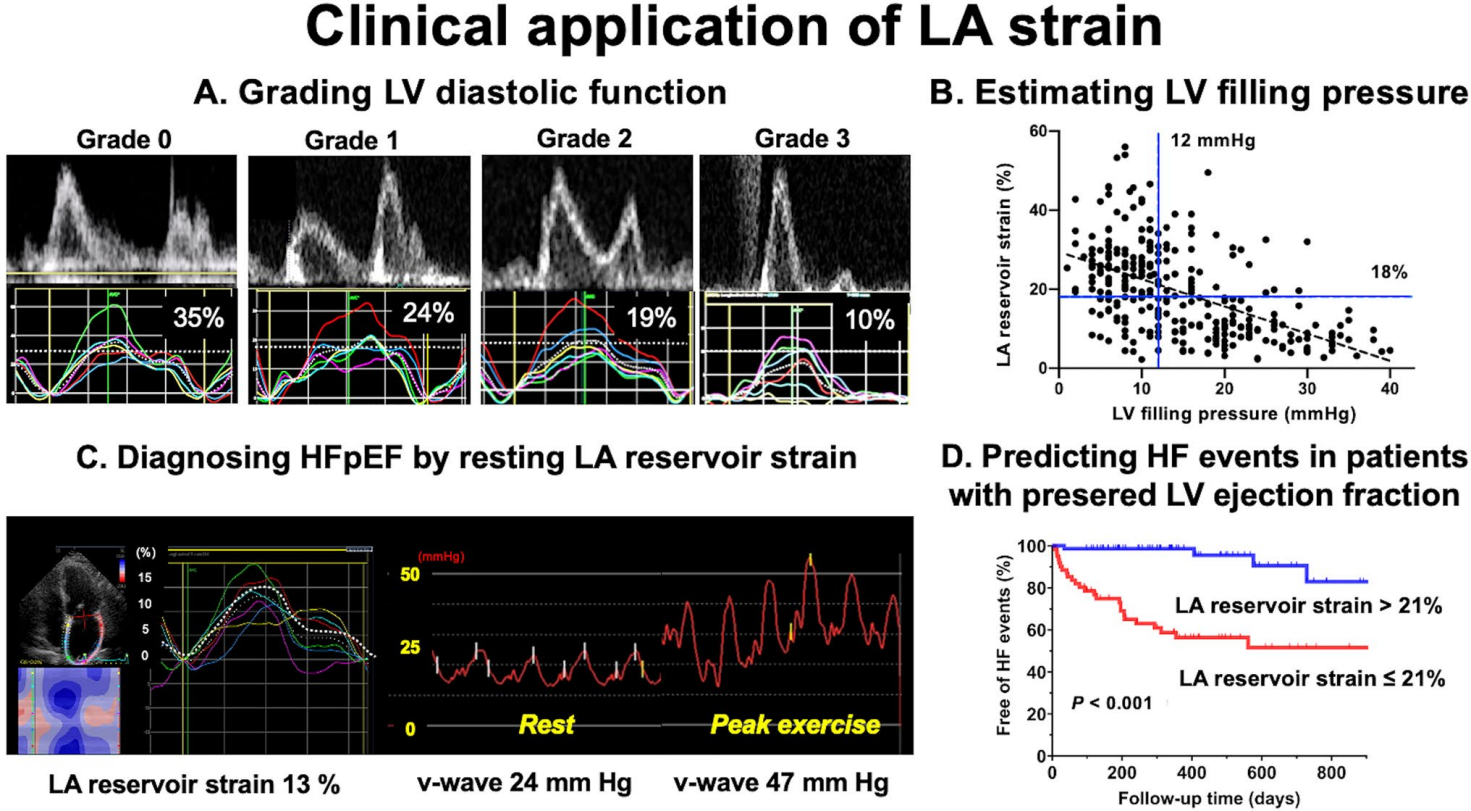

**Supplementary Information:**

The online version contains supplementary material available at 10.1007/s12574-025-00695-x.

## Basic physiology of LA function

Left atrial (LA) function is occasionally underestimated as compared to left ventricular (LV) function in the total role of cardiac performance. However, LA function could compensate to maintain normal cardiac output when LV function fails. Inevitably, the importance of LA function has been recognized, and researchers have investigated LA mechanics and contributions of LA mechanics to cardiac performance.

The LA myocardium contracts to produce LV filling at LV end-diastole after P-wave onset and maintains stroke volume according to the Frank–Starling law [1]. LA booster pump function is governed by LA intrinsic contractility as well as preload and afterload conditions. For example, LA booster pump function is augmented to expand a hypertrophied ventricle to produce normal stroke volume in patients with hypertensive LV hypertrophy. The hypertrophied LV wall is histologically produced by muscular hypertrophy and interstitial fibrosis, which are associated with an afterload increase in the left atrium.

The mitral valve closes and LA contraction ends in the QRS complex. Subsequently, LA relaxation begins to produce LA early reservoir function as a suction force of effective blood flow from the pulmonary vein to the left atrium. The sum of LA contraction and relaxation corresponds to an active LA function. These two functions are molecularly linked as a role in excitation–repolarization coupling and functionally connected as a mechanistic link between systolic contraction and early diastolic recoil. When atrial fibrillation (AF) occurs, a patient immediately loses these two functions, which contributes to one-third of LV filling, [2]. An augmented LA booster pump and early reservoir function already works to prevent an onset of heart failure (HF) in patients with LV dysfunction. However, AF occurrence leads to overt HF due to the loss of these active LA functions.

LV long-axis shortening produces a pulling force of the mitral annular plane to the cardiac apex during ventricular ejection phase, resulting in LA cavity expansion. Passive LA dilatation corresponds to LA late reservoir function. Thus, LA reservoir function is tightly coupled to LV longitudinal systolic function [3]. LA compliance has also a significant impact on effective inflow from the pulmonary vein to the left atrium during the LA late reservoir phase. In patients with stiff LA syndrome, the blood inflow is limited due to increased LA pressure during the late reservoir phase, caused by a noncompliant left atrium.

The stored blood in left atrium during the reservoir phase energetically drains into the left ventricle due to LV relaxation. Left atrium works as a conduit function that contributes to LV filling [4]. The relative importance of conduit function is dependent on LA compliance. As the left atrium becomes stiffer and its reservoir function is reduced, the importance of conduit function increases [4, 5].

### Echocardiographic assessment of LA function

Echocardiography provides pivotal information about cardiac structural abnormalities, functional impairments, and elevated ventricular filling pressure in patients with HF. LA dilatation is a hallmark in patients with both HF phenotypes of reduced and preserved ejection fraction (HFrEF and HFpEF, respectively). Hence, the LA volume index is generally measured as an echocardiographic marker of the chronic burden of LV diastolic dysfunction and elevated filling pressure [6]. LA volume was measured using the biplane disk summation method using apical four- and two-chamber views by echocardiography [7].

**Fig. 1:**
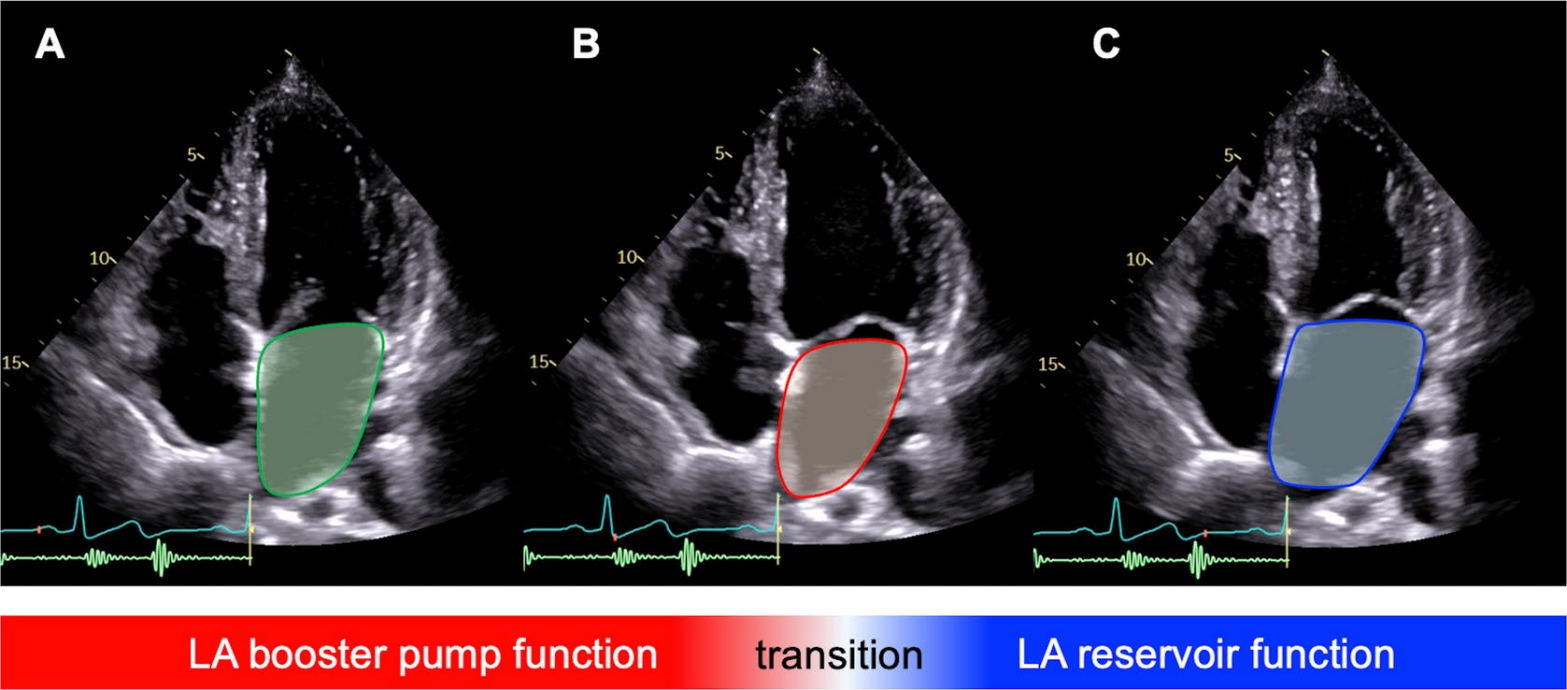
LA volumetric change from the apical four chamber view. Figure shows LA volumetric change from the apical four-chamber view. At the onset of P-wave **A**, the LA myocardium starts to contract for minimizing LA volume at the QRS complex **B**. The volumetric change in this phase (LA active emptying fraction) is a parameter of LA booster pump function. Immediately after LA contraction, the LA myocardium relaxes due to LA active relaxation and passive dilatation to reach the LA maximal volume **C**. The volumetric change in this phase (LA total emptying fraction) is a parameter of LA reservoir function. LA, left atrial

LA dilatation co-exists with its functional impairment. LA function assessed by echocardiography has growing evidence of incremental diagnostic and prognostic values in patients with HF. The LA emptying fraction, which is measured from LA volumetric change, is one of the echocardiographic parameters for LA function. Figure 1 demonstrates LA volumetric change from an apical four-chamber view. The LA volume decreases after P-wave onset to minimize LA volume at the time of mitral valve closure during QRS complex. The volumetric fraction in this phase (LA active emptying fraction) is a parameter of LA booster pump function. Instantaneously, LA volume increases and maximizes just before mitral valve opening. The volumetric fraction in the latter phase (LA total emptying fraction) is a parameter of LA reservoir function. The development of tissue Doppler imaging enables evaluation of myocardial deformation in LV and LA walls. LA longitudinal deformation (LA strain) can be estimated in the LA septal and lateral walls despite methodological limitations due to angle dependency. Speckle tracking echocardiography was introduced to analyze regional myocardial deformation without the angle dependency issue. LA strain by speckle tracking echocardiography has been rapidly utilized for clinical application, especially in patients with HF (Supplementary Figure).

### Measurement of LA strain by speckle tracking echocardiography

Generally, LA longitudinal strain by speckle tracking echocardiography is used in the recent studies to quantify LA function. Because the LA wall is complex and connected to the pulmonary vein and LA appendage, the global value of LA longitudinal strain is interpreted as the length change of the entire LA contour. Although LA strain can be measured in apical four- and two-chamber images, the EACVI/ASE/Industry Task Force recommends using LA strain from apical four-chamber images to increase feasibility for LA strain measurement [8]. It is important to acquire a non-foreshortened LA image to quantify LA strain similar to LA volume measurement [7].

**Fig. 2:**
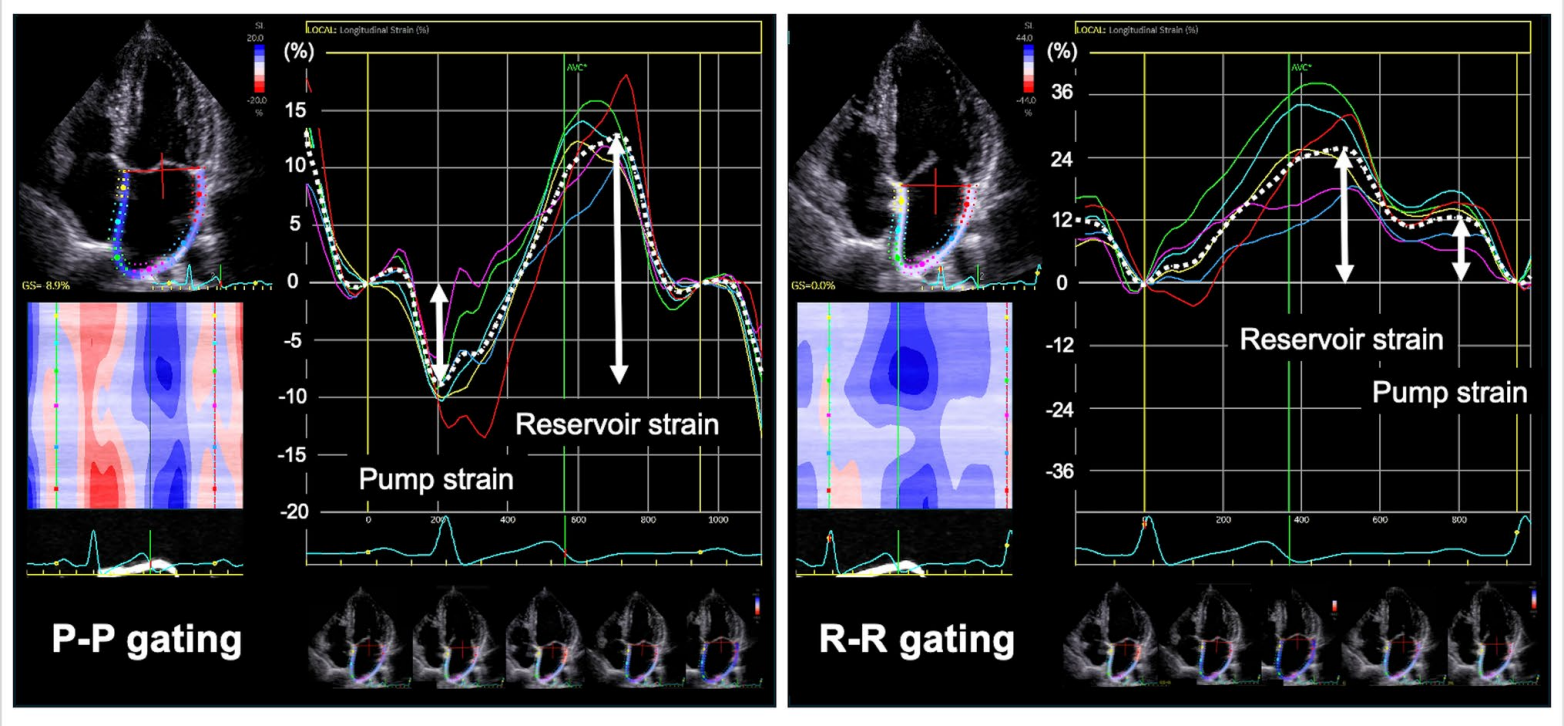
How to measure LA strain. The two methods (P–P and R–R gating) used to measure LA strain are utilized as LA functional assessment. The R–R gating method is generally applied in the clinical setting and research because the R–R gating method can be used to analyze the LA strain, even in atrial fibrillation. LA, left atrial

LA strain can be displayed as the two distinct curves, which are set to zero strain between LA contraction onset and LV end-diastole. The former is represented as the P–P gating method, and the latter is represented as the R–R gating method (Fig. 2). The LA strain curve using the P–P gating method shows a sinusoidal configuration consisting of the first negative wave and the second positive wave. Because the LA myocardium is completely relaxed at P-wave onset, the P–P gating method can help explain a sequence of LA contraction and relaxation corresponding to an intrinsic LA function. After the loss of LA intrinsic function by AF or atrial myopathy, the first negative wave disappears, and the LA strain curve shows the second positive curve only. In contrast to the P–P gating method, the R–R gating method facilitates display of LA strain curve, even in AF rhythm.

**Fig. 3:**
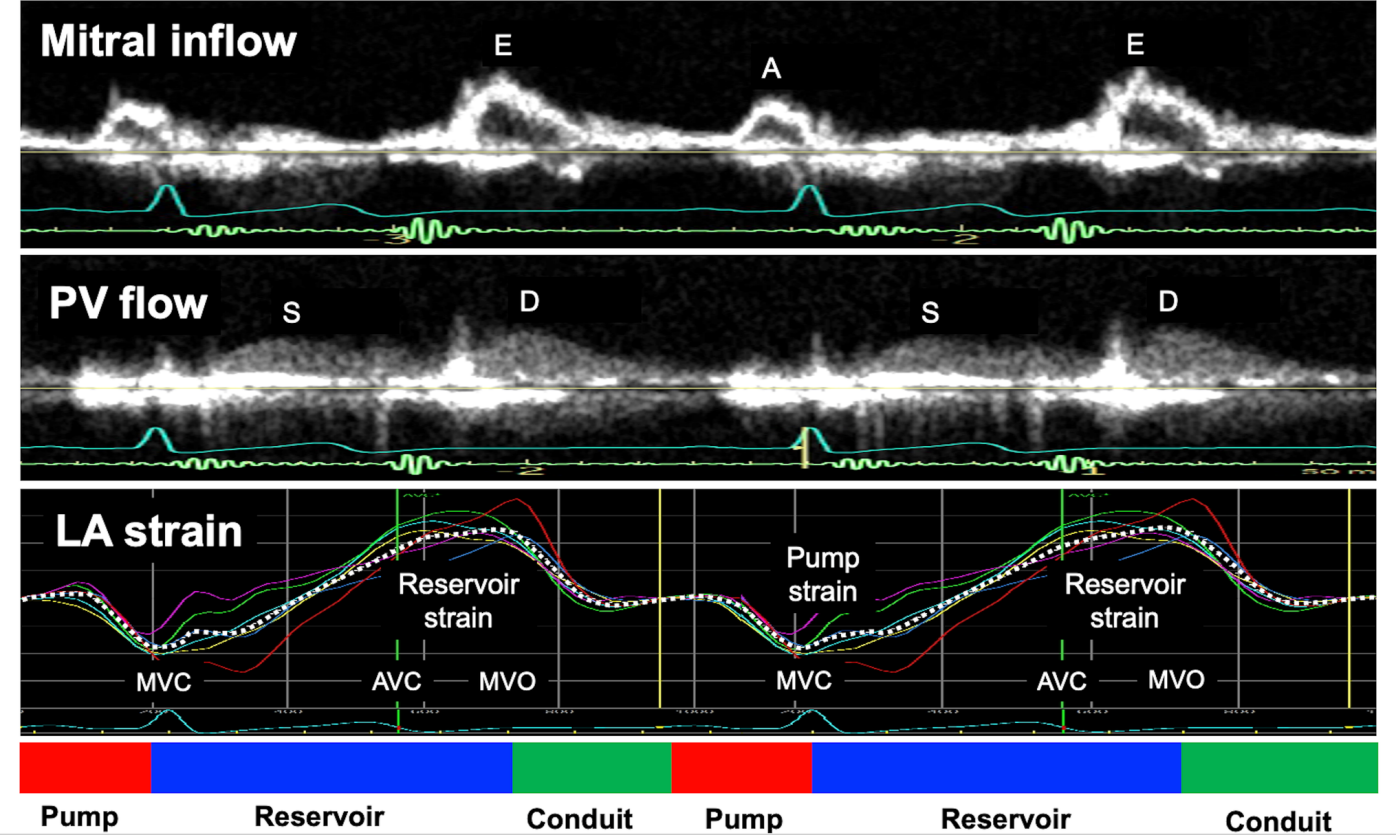
Doppler signals of mitral inflow and pulmonary venous velocities and LA strain curve. The figure demonstrates two Doppler signals of mitral inflow and pulmonary venous flow, as well as LA strain curve simultaneously. LA, left atrial; PV, pulmonary venous; MVC, mitral valve closure; AVC, aortic valve closure; MVO, mitral valve opening

LA strain using the R–R method is available to evaluate LA function with diagnostic value in most patients with HF. Figure 3 shows the LA strain curve using the R–R gating method together with Doppler signals of mitral inflow and pulmonary venous flow.

LA strain components represent reservoir, conduit, and booster pump function. Three parameters of LA strain can be estimated during sinus rhythm using the P–P and R–R gating methods, while LA pump strain cannot be detected during AF. LA reservoir strain value is higher using the R–R gating method than the P–P gating method because the strain value is determined by the baseline length. The length of the LA contour at LV end-diastole is shortened due to LA contraction compared to LA end-diastole at P wave onset.

### Determinants of LA reservoir strain

LA reservoir strain is of great interest among strain parameters for diagnosis and predictive values in HF. It is essential to understand the determinants of LA reservoir strain from the physiological and mathematical perspectives.

The importance of LA reservoir function had been highlighted by notable studies, such as Suga in 1974 [9], Toma in 1987 [10] and Barbier in 1999 [11]. LA reservoir function is influenced by LA contraction, relaxation, LV contraction through descent of the cardiac base during ventricular systole, LA chamber stiffness, and right ventricular (RV) systole through pulmonary venous inflow [11]. Taken together, these findings confirm that LA reservoir function is affected by LA intrinsic function as well as ventricular systolic function.

We previously investigated the determinants of LA reservoir strain in a multicenter study involving 322 patients with cardiovascular disease of different etiologies [12]. The strongest determinants of LA reservoir strain were shown to be LV global longitudinal strain (GLS) and secondary LV filling pressure (Graphical Abstract) [12]. Recently, Mălăescu et al. [3] investigated the major determinant of LA reservoir strain during the cardiac phases. They concluded that a close linear relationship exists between LA reservoir strain and LV GLS, and the slope of the relationship was correlated with LV/LA volume ratios. The study also demonstrated that LA reservoir strain had the potential to predict cardiovascular events of HF hospitalization and new AF occurrence [3].

LA reservoir strain is mechanistically and methodologically coupled to LV GLS. It interrogates not only LA function but also LV systolic and diastolic function, thus potentially adding a prognostic value to cardiovascular outcome despite a single echocardiographic parameter.

### Normal value of LA strain

Because LA structure and function are gradually worsened due to the chronic burden of LV diastolic dysfunction, LA strain can be altered as a parameter of LA function in an aging process [2, 13]. A meta-analysis revealed a normal reference value for LA reservoir strain of 39% (95% confidence interval, 38–41%) and LA pump strain of 17% (95% confidence interval, 16–19%) [14]. The gender difference can also impact the LA strain value because LA size, hemodynamic vulnerability and HFpEF risks are different between genders. Singh et al. [15] recently reported a normal LA strain value. The lower limit of LA reservoir strain is 24% despite a wide range of normal LA strain values between generations and genders, while the LA pump strain value increases with aging.

### Estimating LV diastolic dysfunction and elevated filling pressure by LA strain

Mitral inflow and mitral annular velocities, LA volume index and peak tricuspid regurgitation velocity are utilized for grading LV diastolic function and estimating LV filling pressure from the 2016 ASE/EACVI recommendations [16]. As shown in the Graphical Abstract, LA reservoir strain has an additive value to these conventional parameters in discriminating the grade of LV diastolic function among grades 0, 1, 2, and 3 [17].

LA reservoir function is also associated with LV filling pressure. According to an EACVI expert consensus document, the cutoff value of LA reservoir strain (18%) can be used to differentiate normal or elevated LV filling pressure [18].

Evidence supporting the clinical utility of LA pump strain rather than LA reservoir strain is insufficient. However, it is important to recognize that LA pump strain corresponds to LA intrinsic function and has an important role in maintaining normal LA pressure when LV function fails. According to our previous report, the preservation of LA pump strain (> 14%) was a sign of normal LV filling pressure in patients with a preserved LV ejection fraction [12].

### Clinical application of LA strain in cardiovascular disease

#### HFpEF

HF is a significant public health problem, with a worldwide prevalence of over 64 million [19]. HFpEF accounts for 70% of all HF cases and is becoming the dominant form of HF [20, 21]. The left atrium has a substantially important role in HFpEF by regulating LV filling and cardiac output without congesting the lungs [5, 22]. Traditionally, LA dilation is considered a marker of LV diastolic dysfunction [23]. However, growing evidence has demonstrated that, beyond LA remodeling, LA dysfunction is an important pathophysiologic driver of HFpEF [23], and that LA strain analysis provides valuable information on LA dysfunction, diagnosis, and risk stratification in patients with HFpEF [5, 24, 25]. LA dysfunction is common in patients with HFpEF. LA reservoir and booster pump function assessed by speckle tracking echocardiography are reduced in patients with HFpEF compared to control subjects without HF [22, 26–29]. This finding may be related to LA structural remodeling, AF burden, and increased LA afterload (i.e., elevated LV filling pressure) [12, 27, 29–31]. Abnormal indices of LA strain, particularly reduced LA reservoir strain, are associated with severe symptoms, AF progression, exercise intolerance, and increased risks of clinical outcomes [22, 30, 32].

With the emergence of life-saving pharmacotherapies such as sodium-glucose cotransporter 2 (SGLT2) inhibitors, HFpEF has become a treatable condition [33, 34]. This paradigm shift has made the accurate and timely diagnosis of HFpEF more important than ever before [33, 34]. However, diagnosis remains challenging, especially for individuals presenting with chronic dyspnea [35, 36]. Echocardiography has a key role in HFpEF diagnosis [37]. The American Society of Echocardiography and the European Association of Cardiovascular Imaging (ASE/EACVI) recommend a combination of multiple echocardiographic markers of LV diastolic function but this is reported to have low sensitivity in identifying elevated LV filling pressure with indeterminate cases being common [38–41].

Growing body of evidence has demonstrated the potential usefulness of LA strain, especially LA reservoir strain, to identify elevated LV filling pressure and thus HFpEF. Lower LA reservoir strain strongly correlates with higher LV filling pressure that is measured invasively (*r* = 0.79) [12, 42]. Multiple studies have reported reasonable discriminative abilities of reduced LA reservoir strain for HFpEF from noncardiac causes of dyspnea (area under the curve [AUC], 0.72–0.85) [27–29]. Although further studies are required to develop the optimal cutoff point, these data suggest that LA reservoir strain may help diagnose HFpEF among individuals with unexplained dyspnea.

An advantage of LA reservoir strain is high feasibility, and incorporation into the algorithm can reduce indeterminate cases [12, 18]. Patients with HFpEF often have normal LV filling pressure at rest and the pressure increases only during physiologic stress such as exercise [38, 43]. Hence, echocardiographic markers of LV diastolic function are often normal at rest, making the identification of HFpEF far more difficult [38]. In this context, a study reported a moderate correlation between LA reservoir strain at rest and pulmonary capillary wedge pressure during ergometry exercise (*r* = –0.64, *P* < 0.001), suggesting that impaired LA reservoir strain may predict abnormal hemodynamics that only develop during exercise in HFpEF [28].

Exercise stress echocardiography is useful for identifying occult HFpEF by estimating increases in intracardiac pressures during exercise, and has been reviewed in detail elsewhere [27, 37, 38, 44–46]. Normal left atrium dilates during dynamic exercise to augment LA reservoir function and receive increased venous return from the pulmonary veins without a significant increase in LA pressure. When LA dysfunction develops with decreasing compliance in HFpEF, the ability to function as a reservoir chamber may be limited, leading to a marked elevation in LA pressure during exercise (Graphical Abstract) [47, 48]. Therefore, assessment of LA reservoir strain during exercise (i.e., response to exercise) may help diagnose HFpEF. Recent studies have demonstrated that increases in LA reservoir strain are lower in patients with HFpEF than noncardiac dyspnea with an increase in the E/e’ ratio during exercise [22, 27]. Thus, exercise LA reservoir strain provides an excellent diagnostic performance for HFpEF (AUC, 0.80; *P* < 0.0001) [27]. When determining “LA compliance” by the ratio of LA reservoir strain to E/e’ ratio, exercise LA compliance demonstrated an excellent ability to diagnose HFpEF (AUC, 0.87; *P* < 0.0001) compared to the exercise E/e’ ratio, exercise LV GLS, or exercise LA reservoir strain alone [27]. Importantly, lower exercise LA reservoir strain has been shown to be associated with reduced exercise capacity and worse clinical outcomes in patients with HFpEF (Graphical Abstract) [22, 46].

**Fig. 4:**
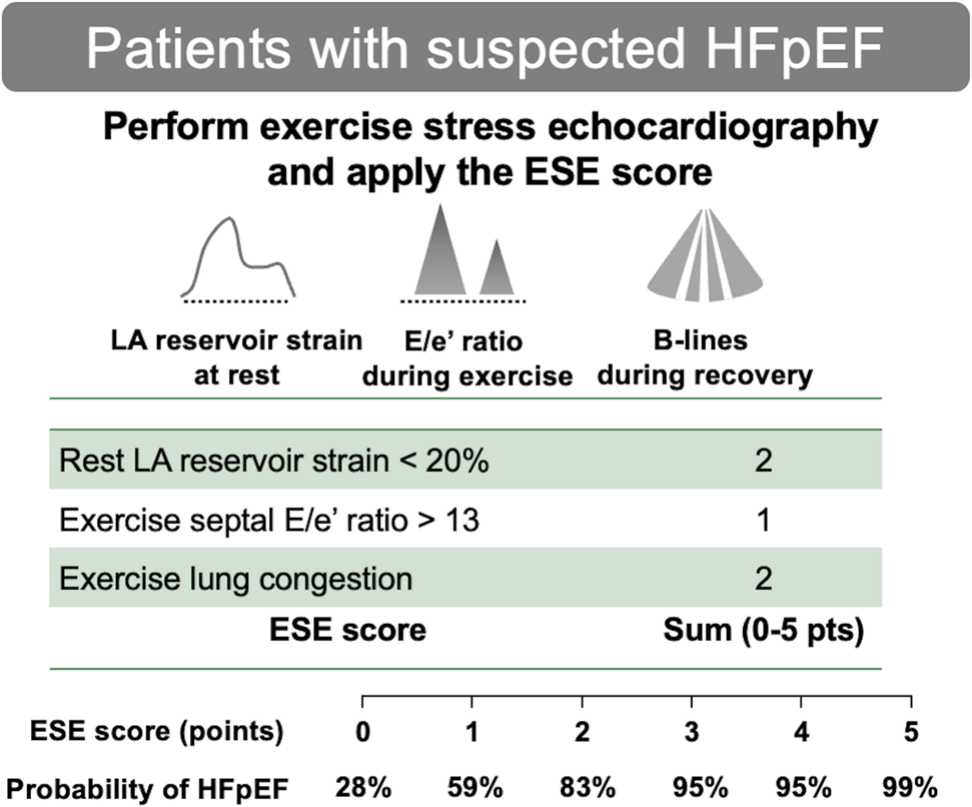
A proposed diagnostic approach for HFpEF using the ESE score. The ESE score is calculated by LA reservoir strain at rest, early diastolic mitral inflow-to-mitral annular tissue velocity (E/e’) ratio during peak ergometer exercise, and development of lung congestion during the recovery period. In patients with intermediate pre-test probability of HFpEF, exercise stress echocardiography should be performed. The ESE score can be applied to estimate post-test probability. HFpEF, heart failure with preserved ejection fraction; ESE, exercise stress echocardiography; LA, left atrial

Despite the increasing evidence supporting the clinical utility of exercise stress echocardiography, the primary limitation is the lack of universal diagnostic criteria [16]. Although some societies propose algorithms to diagnose HFpEF based on the exercise stress echocardiography, these are based on the expert opinion [16, 49]. This approach leads to diagnostic uncertainty or discrepancy, resulting in under-diagnosis of HFpEF and ultimately a lack of appropriate treatment in clinical practice [35, 41]. An evidence-based weighted scoring system, exercise stress echocardiography score (ESE score), was recently developed with case definition ascertained by the gold standard of exercise right heart catheterization [50]. The ESE score incorporates 3 echocardiographic variables: exercise E/e’ ratio > 13 (1 point); exercise lung congestion (2 points); and resting LA reservoir strain < 20% (2 points). This score demonstrated strong discrimination of HFpEF from noncardiac dyspnea with a superior diagnostic ability to the proposed criteria from the ASE/EACVI (AUC, 0.90 vs. 0.66; *P* < 0.0001). The probability of HFpEF significantly increases as the ESE score increases (28% probability for an ESE score = 0, 59–83% for an ESE score = 1–2, and 95–99% for an ESE score ≥ 3; Fig. 4) [50]. The ESE score may help diagnose HFpEF among individuals presenting with dyspnea in clinical practice based on Bayesian theory. First, assessments of pre-test probability will be performed based on the clinical demographics, physical examination findings, natriuretic peptide level, chest-X ray, and rest imaging. Pre-test probability can be also estimated by the H_2_FPEF score or the HFA–PEFF algorithm. Then, exercise stress echocardiography should be performed on patients with intermediate pre-test probability and the ESE score can be applied. If a patient with an H_2_FPEF score of 2 points (40% pre-test probability) had an ESE score of 0 points (low probability, negative likelihood ratio = 0.1), the post-test probability would decrease to 7%, and HFpEF could be excluded with a high degree of confidence. In contrast, an ESE score > 3 points (high probability, positive likelihood ratio = 2.6) in a patient with an H_2_FPEF score of 4 points (pre-test probability = 70%) would increase the post-test probability to 97%, providing a definitive diagnosis of HFpEF. Invasive stress testing may be considered to confirm the diagnosis in patients with an intermediate post-test probability. Further studies are needed to determine the optimal approach for evaluating and managing HFpEF using LA strain analysis.

### Atrial fibrillation

AF is the most common arrhythmia in elderly populations and can trigger the occurrence of HFpEF. AF onset promotes an increased heart rate with an irregular rhythm, resulting in inefficient ventricular filling and tachycardia-induced cardiomyopathy. A compensatory mechanism of LA active function including LA contraction and relaxation, operates to maintain cardiac output in asymptomatic patients with LV dysfunction. The loss of LA active function eventually results in the transition from preclinical HF (stage B) to clinical HF (stage C). The LA contribution during afterload increase was experimentally examined; results indicated that LA active function immediately works to maintain stroke volume and normal LA pressure despite LV systolic and diastolic dysfunction after an increase in central blood pressure created by aortic banding [51]. In patients with AF or atrial remodeling, increased central blood pressure can trigger HF development due to loss of LA active function.

Catheter ablation is an established therapy to maintain sinus rhythm in patients with AF, while it is challenging to treat AF by ablative therapy in patients with advanced LA remodeling or significant valvular regurgitation. The strategy of AF ablation should be discussed for medical and interventional therapies or supportive care based on lifelong management in each AF patient.

### Hypertrophied LV

Increased LV wall thickness is one of the representative echocardiographic findings in patients with HFpEF. Hypertension and aortic stenosis pose a burden on the left ventricle through pressure overload, thus promoting LV concentric hypertrophy. In the absence of hemodynamic unloading by pharmacological or interventional therapy, LV systolic and diastolic function could worsen due to cardiac muscular hypertrophy and interstitial fibrosis. In patients with LV hypertrophy, LV longitudinal shortening (i.e., LV GLS) is already reduced in parallel with reduced LA reservoir function despite a preserved LV ejection fraction [52].

Hypertrophic cardiomyopathy and cardiac amyloidosis are the two different etiologies presenting as an increased LV wall thickness. LA pump function is augmented to expand a stiff left ventricle, thus facilitating stroke volume maintenance in patients with hypertrophic cardiomyopathy. These patients are sometimes asymptomatic at rest if LV obstruction and massive LV hypertrophy are not present. In contrast, patients with cardiac amyloidosis easily develop symptoms and signs of HF because abnormal amyloid fibrils can be deposited in the atrial and ventricular walls. Amyloid deposition in the left atrium leads to LA myopathy and chamber stiffening. Patients with cardiac amyloidosis have significant risks of refractory HF as well as thromboembolic events, even in sinus rhythm.

It is generally accepted that the relative apical sparing patterns of LV longitudinal strain help diagnose cardiac amyloidosis with better precision than conventional parameters [53]. The relative apical sparing pattern is a strain distribution in which LV strain at the basal wall is reduced despite preserved LV strain at the apex. Previously, Barbier et al. reported that LV basal descent, which is produced by LV long-axis shortening, is one of the major determinants of LA reservoir function [11]. Based on our recent data, LV longitudinal strain at the cardiac base is associated with LA reservoir strain in patients with cardiac amyloidosis rather than other LV hypertrophy etiologies. Therefore, LA reservoir strain can provide additional information to the apical sparing pattern in discriminating between the cardiac amyloidosis etiology and other LV hypertrophy etiologies [52].

### Stiff LA syndrome

Patients with a stiff LA syndrome have been historically recognized to develop HF symptoms, particularly among patients with mitral valve stenosis or prosthetic mitral valve replacement. Computed tomography imaging occasionally reveals LA wall calcifications, which are associated with LA chamber stiffening in these patients [54, 55].

LA stiffness is physiologically calculated as a change in LA pressure with a change in LA volume in the reservoir phase when the mitral valve closes. LA stiffness is estimated from an ascending slope on the LA pressure–volume loop if instantaneous measures of LA pressure and volume are available [11]. Cardiac amyloidosis is a representative disease where stiff LA syndrome develops due to abnormal amyloid deposition in the left atrium. Echocardiographic findings, such as decreased Doppler flow velocity of pulmonary venous S-wave and low value of LA reservoir strain, are characteristic of indicating LA stiffening in patients with cardiac amyloidosis. The supplementary movie demonstrates a case of cardiac amyloidosis with LA stiffening by comparing a case with preserved LA function.

Stiff LA syndrome is currently being revisited in patients with AF treated by repeated catheter ablation [56]. According to a recent report, LA wall calcification was observed in 14% of patients who had undergone AF ablation. These patients experienced more cardiovascular events compared to patients without LA wall calcification [57]. Exertional dyspnea is one of the representative symptoms in AF patients with stiff LA syndrome. These patients often have extensive and irreversible injuries in the left atrium, resulting in AF recurrence and refractory HF. It becomes increasingly important to objectively diagnose LA structural and functional remodeling in AF patients. LA strain is a potential indicator of LA myopathy or stiffening in these patients.

### Pulmonary hypertension

It is crucial to estimate the probability of pulmonary hypertension (PH) in patients with cardiac or noncardiac dyspnea. Among echocardiographic parameters, the peak velocity of tricuspid regurgitation (TRV) has a central role in diagnosing the probability of PH.

Because the presence or absence of PH cannot be reliably determined by TRV alone, other echocardiographic signs suggestive of PH should be evaluated: RV dilatation; interventricular septum flattening; shortened acceleration time or notching of the RV outflow tract Doppler velocity waveform; dilatations of inferior vena cava and right atrial area; and RV systolic dysfunction [58].

It is important to evaluate whether PH is induced by pulmonary artery disease (pre-capillary PH) or left-sided heart disease (post-capillary PH) when assessing probable PH patients. Because there is a distinct difference in LA pressure between the two entities (normal LA pressure in pre-capillary PH and elevated LA pressure in post-capillary PH),

LA reservoir strain can be used to differentiate the two entities because LA reservoir strain is a mirror of LV systolic and diastolic function [59]. We previously investigated 110 patients with probable PH based on a TRV > 2.8 m/sec and/or other PH signs. The mitral E/A ratio in combination with LA reservoir strain facilitated the differentiation of patients between pre-capillary PH (preserved LA strain ≥ 16%) and post-capillary PH (low LA strain < 16%) with an accuracy of 85% [60].

## Conclusions

Clinical application of LA strain is helpful in assessing LV diastolic dysfunction, estimating elevated LV filling pressure at rest as well as during exercise, and predicting future outcome in patients with HF. Although it is still challenging to accurately assess LV diastolic function by echocardiography, incorporating LA strain analysis could provide a new perspective to unveil the mysteries of LV diastolic function.

## Supplementary Information

Below is the link to the electronic supplementary material.

Supplementary file1 (MOV 8284 KB)

Supplementary file2 (TIFF 16063 KB)

## Data Availability

The data sharing is not applicable to this review article as no new data were created.
