# Supplementary figures and images for: Clinical utility of the left atrial strain analysis
Source: J Echocardiogr. 2025 Jul 3;23(3):145–55. doi: 10.1007/s12574-025-00695-x (PMC12378494; doi:10.1007/s12574-025-00695-x)

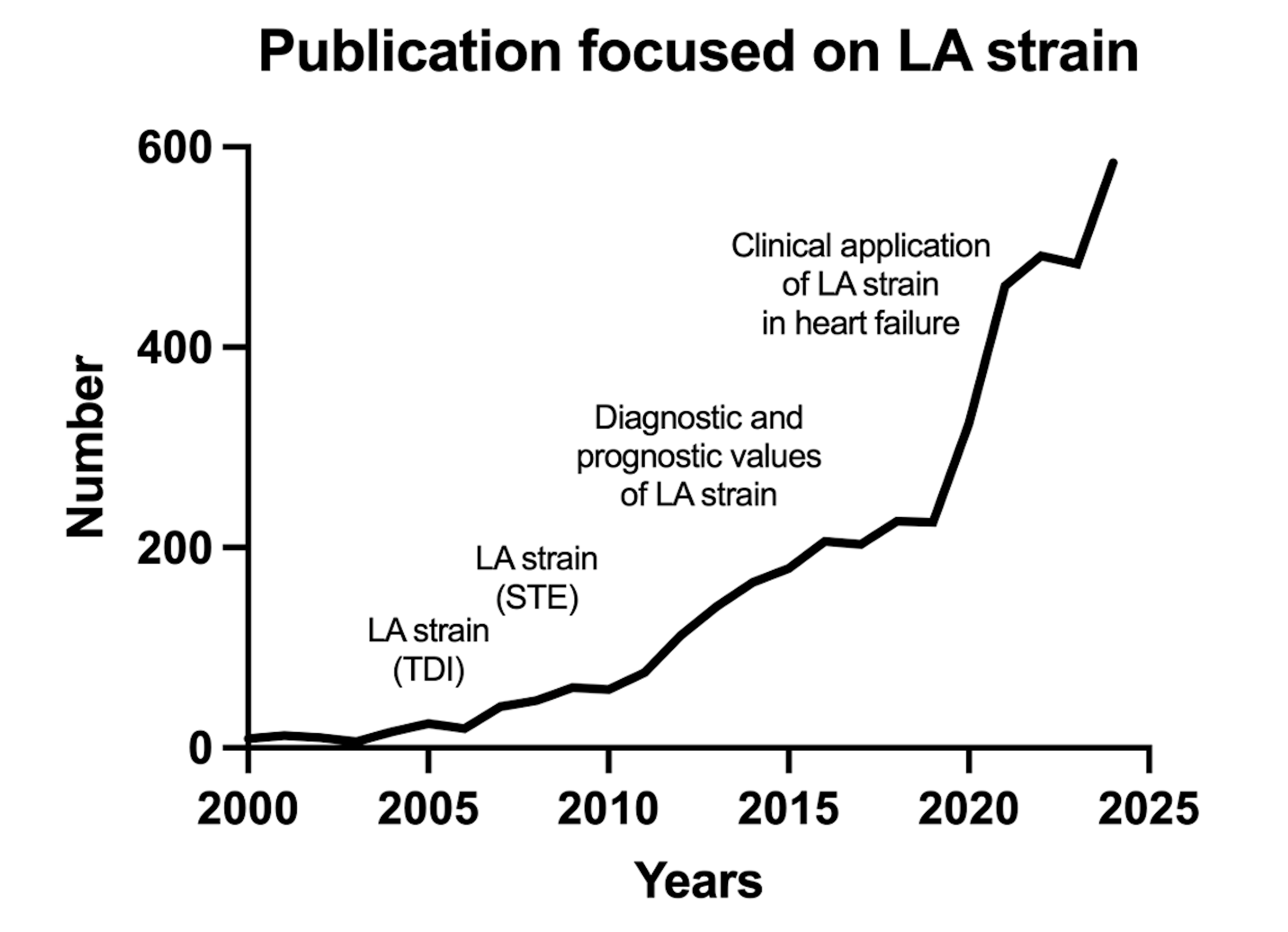

Supplement: Supplementary file 2 — Supplementary file2 (TIFF 16063 KB) [file 12574_2025_695_MOESM2_ESM.tiff]
